# Autonomous Thermal Vision Robotic System for Victims Recognition in Search and Rescue Missions

**DOI:** 10.3390/s21217346

**Published:** 2021-11-04

**Authors:** Christyan Cruz Ulloa, Guillermo Prieto Sánchez, Antonio Barrientos, Jaime Del Cerro

**Affiliations:** Centre for Automation and Robotics (CAR) Universidad Politécnica de Madrid—Consejo Superior de Investigaciones Científicas, 28006 Madrid, Spain; guillermo.prieto.sanchez@alumnos.upm.es (G.P.S.); antonio.barrientos@upm.es (A.B.); j.cerro@upm.es (J.D.C.)

**Keywords:** robotic systems, thermal images, convolutional neural networks, computer vision, search and rescue robots, ROS, Unitree A1

## Abstract

Technological breakthroughs in recent years have led to a revolution in fields such as Machine Vision and Search and Rescue Robotics (SAR), thanks to the application and development of new and improved neural networks to vision models together with modern optical sensors that incorporate thermal cameras, capable of capturing data in post-disaster environments (PDE) with rustic conditions (low luminosity, suspended particles, obstructive materials). Due to the high risk posed by PDE because of the potential collapse of structures, electrical hazards, gas leakage, etc., primary intervention tasks such as victim identification are carried out by robotic teams, provided with specific sensors such as thermal, RGB cameras, and laser. The application of Convolutional Neural Networks (CNN) to computer vision is a breakthrough for detection algorithms. Conventional methods for victim identification in these environments use RGB image processing or trained dogs, but detection with RGB images is inefficient in the absence of light or presence of debris; on the other hand, developments with thermal images are limited to the field of surveillance. This paper’s main contribution focuses on implementing a novel automatic method based on thermal image processing and CNN for victim identification in PDE, using a Robotic System that uses a quadruped robot for data capture and transmission to the central station. The robot’s automatic data processing and control have been carried out through Robot Operating System (ROS). Several tests have been carried out in different environments to validate the proposed method, recreating PDE with varying conditions of light, from which the datasets have been generated for the training of three neural network models (Fast R-CNN, SSD, and YOLO). The method’s efficiency has been tested against another method based on CNN and RGB images for the same task showing greater effectiveness in PDE main results show that the proposed method has an efficiency greater than 90%.

## 1. Introduction

According to a report by the UNDRR (United Nations Office for Disaster Risk Reduction) [1], 7348 natural disasters have occurred in the last two decades. Most of them are caused by floods, storms, and earthquakes, accounting for 40%, 28%, and 8%, respectively [2]. In disasters such as major storms, fatalities occur in ~10% of those affected, while in earthquakes, the number of fatalities is ~49%.

In these scenarios, robotics helps the speed and efficiency of their management [3,4,5]. Search and rescue robots specialized in victim recognition and search are designed to assist in locating victims who are out of sight or in inaccessible locations [6,7]. In addition, the use of these robots also prevents operators from exposing themselves to hazards such as landslides [8,9,10].

In recent years, with the rise of Machine Vision due to new techniques which employ neural networks for tasks such as object classification and detection in images and videos, their application in multiple areas has been explored and investigated. One of these areas is thermography [11,12], some applications such as surveillance and face recognition [13,14,15,16,17,18] have been developed.

There is also a branch of research on people detection with very low-resolution thermal cameras or even from infrared sensors [19,20,21,22]. Note that some works are currently emerging, such as the one by Perdana et al. [20], in which the first steps are taken in the victim detection using neural networks and thermal imagery.

The main developments for people detection in Search and Rescue scenarios using robots, focus on using RGB cameras (additive color mode Red, Green, and Blue) [23,24,25]. The methods based on thermal images are very limited to classical computer vision techniques applications and primitive neural networks. In addition, data acquisition is performed in open areas or using drones for data capturing in daytime conditions [26,27,28,29,30].

The TASAR (Team of Advanced Search And Rescue Robots) project focuses on using terrestrial Search and Rescue Robots for Humanitarian Assistance and Disaster Relief (HA-DR missions) [31]. A robotic system was implemented to validate this proof of concept. It uses the Robot Unitree A1 robot equipped with a sensory system (Thermal Camera Optris PI640, Real-Sense, RPLidar) for data capture and transmission. This quadruped robot was used due to its remarkable adaptability to move in unstructured environments [32].

The main contribution of this work focuses on developing an integrated system, which uses the Unitree Robot (teleoperated) inside a PDE, capable of transmitting data (thermal images, RGB images) for its processing in real-time; in an early detection phase it checks for the presence of victims within the environment. The location of victims is executed by processing the thermal images through a Convolutional Neural Network—YOLO (You Only Look Once)—to later issue an alert and generate a trace with the location of the victim. After a comparative experimental phase between the Faster R-CNN, SSD, and YOLO networks, the YOLO network model has been defined as the most efficient model.

This method was contrasted with conventional RGB image methods, using another trained neural network (with an RGB dataset captured simultaneously with the thermal image dataset) capable identify victims in PDE. However, in scenarios with bad lighting conditions or obstructive materials, its efficiency is low concerning the proposed thermal method.

The tests were performed to detect victims in environments with poor light conditions (both day–night and indoors–outdoors) and use different materials to cover the victims. The main results show an efficiency of the system upper than 90% in the early detection of victims in PDE.

The algorithms execution and information flow between the field robot, sensors, and the central control computer has been developed using ROS.

This paper is structured as follows. In Section 2, the materials and methods used in this work are introduced in detail, followed by the Results and Discussion in Section 3. To conclude, Section 4 summarizes the main findings.

## 2. Materials and Methods

### 2.1. Materials

**Figure 1:**
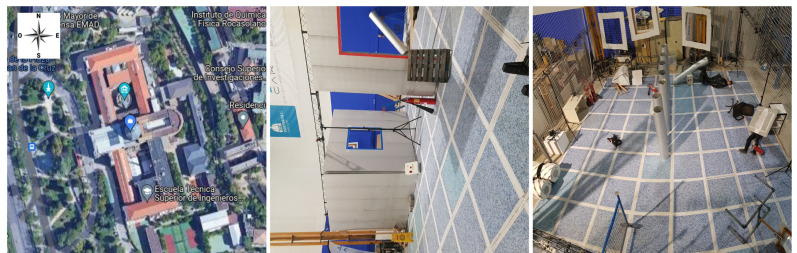
Indoor and outdoor scenarios are used for test development. (**a**) ETSII-UPM Outdoor Testing Environment. (**b**) Scenarios recreated for indoor testing. (**c**) Scenarios recreated for indoor testing—top view. Source: Authors.

The experiments for the development of this research were carried out at the facilities of the Centro de Automática y Robótica located in (40${}^{\circ }$26${}^{\prime }$23.4${}^{\prime \prime }$ N–3${}^{\circ }$41${}^{\prime }$21.7${}^{\prime \prime }$ W) as shown in Figure 1, both in outdoor (Figure 1a) and indoor (Figure 1b,c) scenarios. The latter has been recreated based on the NIST (National Institute of Standards and Technology) standardized environments for disaster environments; specifically in the yellow zone (debris and moderate obstacles) [33].

Table 1 shows the equipment used for this research development.

**Figure 2:**
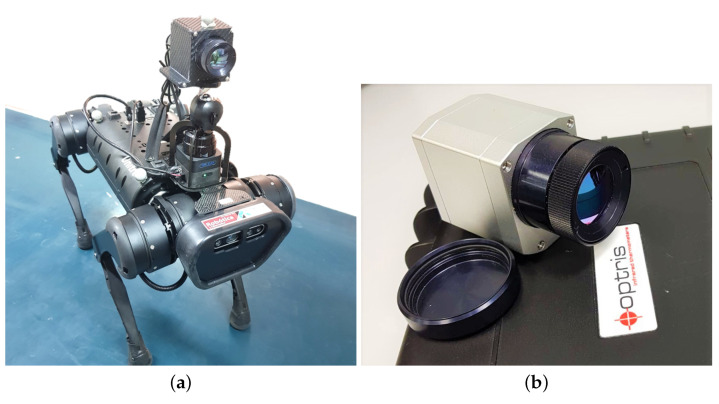
Robot and instrumentation used for the proposed method validation. (**a**) Unitree A1 Robot equipped with thermal camera and Real-Sense. (**b**) Optris Pi640 Thermal Camera.

The robotic system used for the autonomous detection of victims by means of thermal images is mainly integrated by the Unitree A1 robot (Figure 2a) which has integrated into its front part a Real-Sense and the thermal camera (Figure 2b) coupled by means of mechanical support made of a 3D-printed coupling capable of absorbing vibrations emitted due to the robot’s movement due to a ball joints and springs system.

For the processing of the neural network, robot control, and data flow, an MSI computer with an Intel i10 processor and NVIDIA GEFORCE GTX 1660Ti GPU has been used, which supplies the process for its execution in real-time. The computer has been connected by a 5G Wireless Network with the Robot in the field through the ROS Master–Slave (PC MSI-Unitree Robot) Communication System. Real-time requirements were managed by the controller_manager packets, obtaining a latency of 10 ms. The interface used for information management was RVIZ (ROS Visualization).

The infrared camera model Optris PI 640 of the company OPTRIS was used to obtain thermal images. This camera operates in the spectral range of 8 to 14 µm, being able to detect temperatures within the range of −20 to 900 ${}^{\circ }$C; it has a weight of 320 g. It has a thermal sensitivity of 75 mK and an accuracy of ±2 ^∘^C or ±2%, whichever is greater.

The robot used (Unitree A1 of the company UNITREE) has been selected due to its great versatility, agility, and ability to move in environments with unstructured floors (slopes, slopes, debris, etc.), thanks to its system of locomotion by legs, sensors, and real-time processing. The main features of this robot are a maximum speed of 3.3 [m/s], autonomy of one to two hours, great stability, 12 degrees of freedom (DOF), integrated sensors (RPLidar, Real-Sense, IMU, and pressure sensors on the legs), and 3 on-board computers.

### 2.2. Interaction between Subsystems

**Figure 3:**
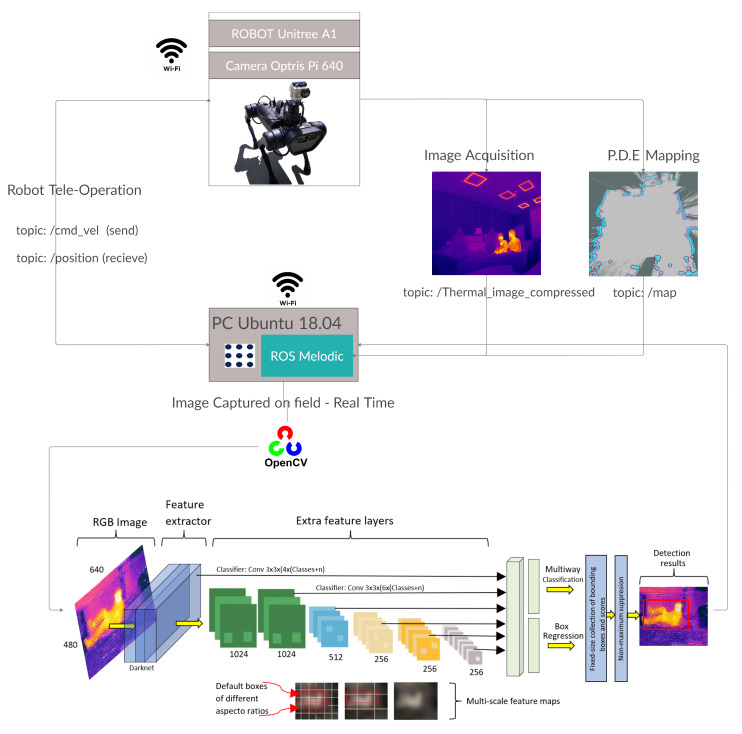
Subsystems integration for the detection of victims in PDE. Source: Authors.

The communications architecture of the implemented method (Figure 3) with the robot in the field, the remote station, and the data processing has been developed as part of the TASAR project entirely using ROS.

The overall system consists of two subsystems, shown in Figure 3: the first one is a high-power central computer that sends the velocity commands to the robot in the field to execute the displacements along the PDE. On the other hand, it receives the position data, thermal, and rgb images from the robot to process them through a convolutional neural network in real time.

The second subsystem is the robot in the field, which thanks to all the instrumentation is in charge of collecting images to send them and, by receiving speed commands, to move through the PDE. The robot in this experimental phase of the system is tele-operated from a remote station by a operator or a previously trained rescuer, sending speed peaks to the robot (/cmd_vel).

Thermal image reception and processing are performed in real-time at the remote station, so that the operator can know beforehand if there is a victim (even if trapped in debris) in need of primary assistance and its location within the environment based on the position of the robot.

For the capture and subsequent transmission of thermal images from the camera integrated in the robot, the drivers for ROS (in the Jetson NX), developed by the manufacturer Optris, have been installed. The method based on fuzzy filters has been used to eliminate noise in thermal images as it is the most recommended for thermal images instead of the conventional median filter methods [34].

For real-time processing, it has been previously configured in the Anaconda virtual environment with the ROS and OpenCV packages, so that, with the network already trained, the inference can be performed. The neural network architecture used is also detailed (this neural network methodology has been applied in other developments such as precision agriculture for detecting fruits in highly noise-contaminated environments [30]). This process can be detailed in the lower part of the Figure 3.

### 2.3. Materials and Termography

This section is focused on highlighting the importance of materials and their interaction and influence on thermal images. It seeks to take advantage of the emissivity of some materials such as plastic or others in order to identify victims that are fully covered.

The fraction of the incident energy that must be captured by the thermal camera to measure the temperature of an object is the emitted energy. Emissivity plays a fundamental role in estimating this energy based on the energy received. Different materials have different emissivity values, leading to more or less accurate temperature measurements. This dimensionless value is quantized from [0–1].

Organic materials generally have a high emissivity, so their measurement is usually straightforward. Materials such as paper, ceramics, wood, soil, plants, sand, rubber, stone, paints, dark or matte coatings, etc. have an emissivity of ~0.95 in the spectral range of 8 to 14 μm. For urban, building, and industrial environments, there are differences in emissivity between different common materials. For building materials such as concrete (0.93), normal brick (0.92–0.94), glazed brick (0.94–0.89), glass (0.95–0.98), and asphalt (0.98), the emissivity is very high and thus the temperature is easily measurable. Table 2 shows the emissivity measurements for different materials.

**Figure 4:**
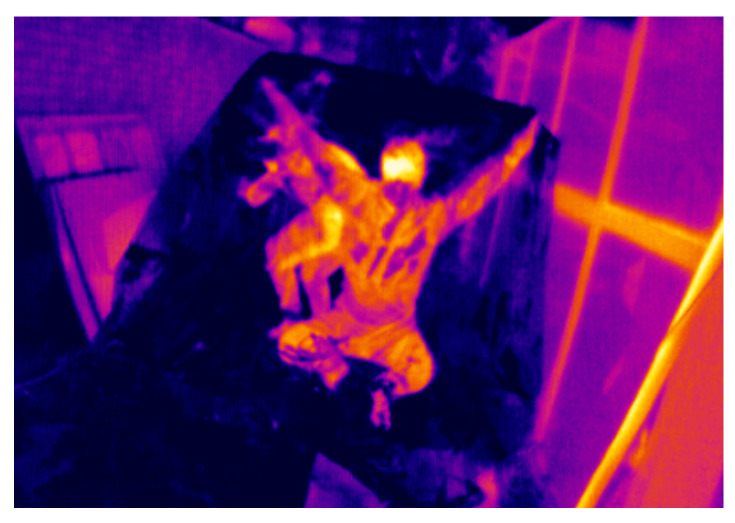
Example of thin-film transmissivity. Source: Authors.

For plastics, the thickness of the object is a significant factor. Thick plastics usually have a high emissivity of approximately 0.86–0.95. The problem arises with thin plastic films. These have a very high transmissivity, so that when measuring the temperature with a temperature camera, what is shown is not the temperature of the film itself, but that of the objects placed behind it, as shown in the Figure 4.

### 2.4. Neural Networks and Environments

One of the main objectives of this work is to compare the performance and accuracy in detecting victims of a number of architectures implementing neural networks. Three networks have been chosen: Faster R-CNN [37], SSD [38], and YOLOv3 [39]. This YOLO version has been used to optimize computational efficiency and achieve real-time development (under the conditions the experiments have developed), avoiding latency in processing. The hyperparameters used for this proposal are Initial Learning rate: 0.001, Learning Rate Schedule: (burn_in = 1000, steps = 400,000, scales = 0.1), Batch size: 16, and Training Epochs: 100.

Each neural network has a number of advantages and disadvantages that distinguish one over the other in different areas, so it is the subject of study to find out which one provides the best characteristics for victim detection.

For the development of this research, multiple virtual environments were set up in Python using Anaconda [40]. Multiple packages such as Tensorflow [41], Pytorch [42], and Open CV [43] were configured and installed in these environments, depending on the network.

For the Faster R-CNN and SSD networks, the Tensorflow library was used using the object detection API developed by Google [44]. For the YOLOv3 network, the pytorch library was mainly used.

### 2.5. Data Collection and Datasets

To train the neural networks, multiple videos were recorded to obtain their corresponding frames in the form of images. Appendix A contains a sample of the generated datasets.

The different Datasets generated for the training have been generated, including images that contain victims under different circumstances, such as partial coverage due to rubble and different materials such as wood or concrete.

It is of utmost importance to obtain robust models by making recordings that show a wide variety of situations and facets.

This is why videos have been recorded in which simulated victims appeared in different positions, at different times of the day, and in which the victim’s body was not shown in its entirety, but only parts of the body. It is of utmost importance to obtain robust models by making recordings that show a wide variety of situations and facets.

Furthermore, the victim’s entire body was not shown, but rather specific body parts such as heads, legs, arms, etc. In addition, materials typical of different environments, such as building materials, were used. Different datasets for the training were generated, including images that contain victims under different circumstances, such as partial coverage due to rubble and different materials such as wood or concrete.

Different environments, such as building site, or urban environment materials, such as metal and wood plates or plastic and fabric films, were used to both simulate the environment and to partially or totally cover the bodies.

### 2.6. Tests and Experiments

**Figure 5:**
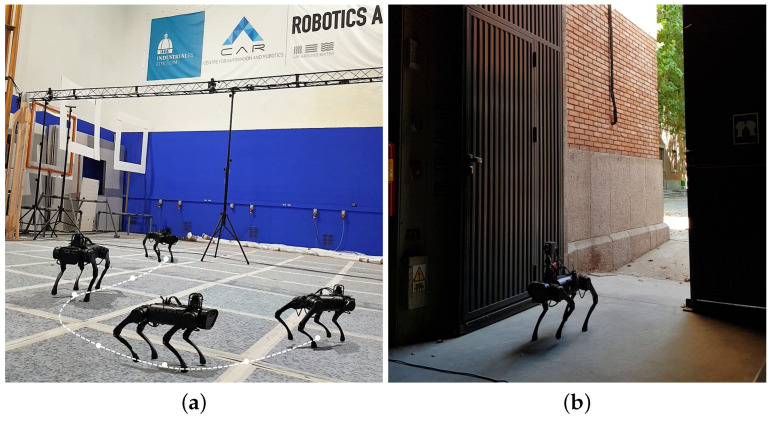
Robot Unitree in different scenarios. (**a**) Robot in indoors (good light conditions). (**b**) Robot in outdoors (bad light conditions). Source: Authors.

As previously mentioned, each neural network has a series of advantages and disadvantages concerning others, such as the precision and speed. A series of tests have been devised in order to corroborate some theoretical aspects and to find the network that works best for victim detection. Figure 5 shows the Unitree Robot in different scenarios during the execution of the field tests. Appendix B shows the videos of the robot’s movement in the scenarios.

**Figure 6:**
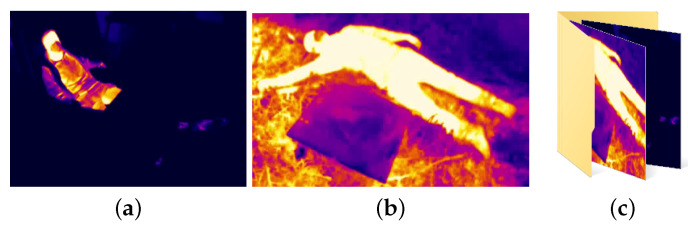
Different datasets used in training. (**a**) Night dataset. (**b**) Day dataset. (**c**) Combined dataset. Source: Authors.

The first test aims to study the influence of temperature contrast on victim detection. This contrast is given between the difference in temperatures captured by the thermal camera of the person or victim to be detected and the environment. At ambient temperatures similar to the average temperature of people outdoors, approximately 20–25 °C, the ambient-person contrast is very low. Therefore, it is intended to test the influence of the thermal contrast on the image by performing tests using three different datasets: one which uses images recorded at night, one which uses images recorded at day, and another which use both of them (both for indoors and outdoors), as shown in Figure 6.

The second test is related to body parts detection. Due to the fact that in accidents or disasters victims may be trapped or buried in debris, the camera may be able to see a single limb or extremity, but not the silhouette of the entire body. Therefore, the networks will be trained so that, apart from detecting people, they can detect different parts of the body. For this reason, in each image of the different datasets, in addition to the ‘person’ label, additional labels will be used: ‘head’, ‘arm’, ‘leg’, and ‘torso’.

The third test consists of distinguishing between rescuers and victims. The most logical method to distinguish rescuers from victims would be to label in the datasets rescuers as ‘rescuer’ and victims as ‘victims’. However, due to the similarity they would have to each other, there would be a very high classification error. A simple method that can be effective is to analyze the dimensions of the bounding box. Accident victims will normally be lying on the ground or in similar positions. This fact can be taken advantage of by measuring the width and length of the of the bounding boxes of the images.

**Figure 7:**
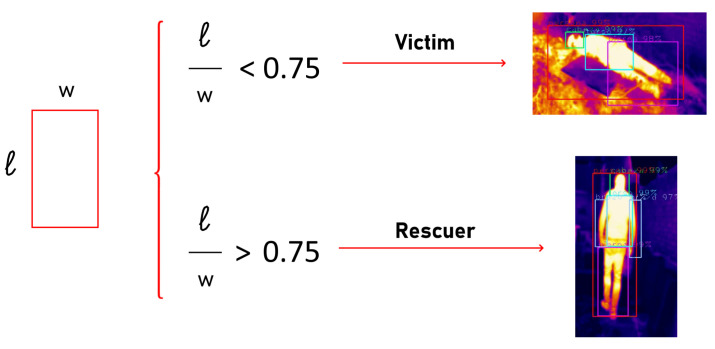
Length-to-width ratio to detect victims. Source: Authors.

Several images showing both rescuers and victims have been analyzed and it has been concluded that the victims have a length-to-width ratio of less than 0.75, as shown in Figure 7.

The proposed method robustness has been contrasted with conventional methods of using RGB images; based on this, different indicators have been obtained, such as detection in night environments, daytime environments, debris, indoor and outdoor environments, and radiation emitted by specific areas. Therefore, the results show that the proposed method relevant to conventional methods.

## 3. Results and Discussion

### 3.1. Analysis of Neural Networks Implemented

#### 3.1.1. Comparison of Faster R-CNN, SSD, and YOLO

**Figure 8:**
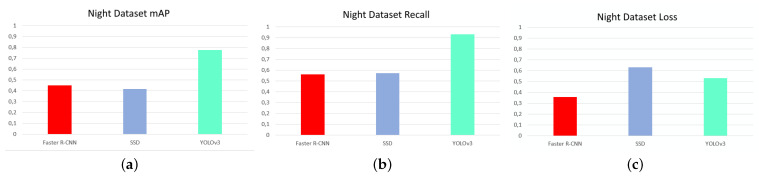
mAP, recall, and loss for networks with night dataset. (**a**) mAP, (**b**) recall, and (**c**) loss. Source: Authors.

**Figure 9:**
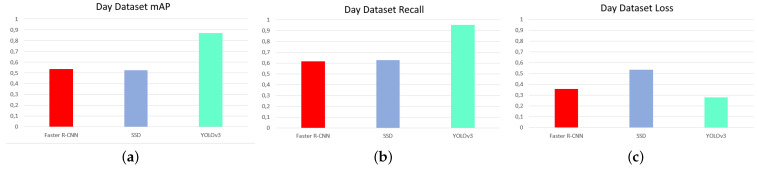
mAP, recall, and loss for networks with day dataset. (**a**) mAP, (**b**) recall, and (**c**) loss. Source: Authors.

**Figure 10:**
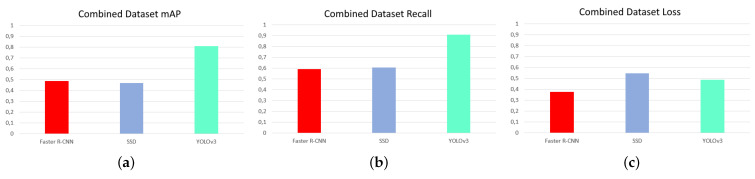
mAP, recall, and loss for networks with combined dataset. (**a**) mAP, (**b**) recall, and (**c**) loss. Source: Authors.

The first test was to measure the influence of temperature contrast on victim detection, with the aim of finding the ideal network and dataset combination. Figure 8, Figure 9 and Figure 10 are shown below, showing the values of accuracy (mAP), recall, and loss for the networks for each dataset.

Analyzing these diagrams it can be seen that the YOLOv3 network is far superior in MAP and recall for all datasets. It also has the lowest loss values of all, except in the case of the night dataset, where the lowest loss is that of the Faster R-CNN network. It follows that, based on evaluation results, the YOLOv3 network is undoubtedly the best of the three.

Another fact to take into account when choosing one network over another is the speed of inference. There is a big difference in the frames per second in inference for each network. The Faster R-CNN network, despite having good precision and recall values and little loss, is extremely slow in inference, working at very few frames per second. The opposite happens for the SSD network, where it does work at a higher fps rate but has a higher loss. YOLOv3 has good values in both the detection parameters and inference speed. There are previous studies that compared the inference execution speed of the networks, such as that in [45], which used a GPU with 12 GB of RAM to compare the performance and speed in inference of multiple networks. The results obtained were an average fps rate for Faster R-CNN of 7, for SSD of 19, and for YOLOv3 of 45.

**Figure 11:**
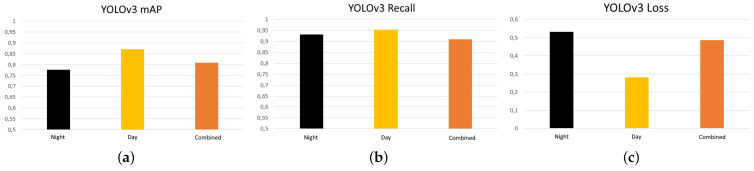
mAP, recall, and loss comparison for the YOLOv3 datasets. (**a**) mAP, (**b**) recall, and (**c**) loss. Source: Authors.

Figure 11 shows the analysis of the YOLOv3 results comparing the datasets.

#### 3.1.2. YOLO Performance with Different Datasets

Figure 11 shows that the highest precision and recall values and the lowest loss values were obtained for the day, combined and night datasets. This is totally contrary to the expected results, which expected that, as there was higher contrast and less influence of other environmental conditions such as solar radiation, the night dataset would be the ideal one. The networks trained with the combined dataset offer values of precision, recall, and loss not much below those of the daytime dataset, with the advantage that they are more versatile, functioning correctly both day and night.

One fact that stands out from the Figure 8, Figure 9 and Figure 10 diagrams is the high loss value that the SSD network has compared to the rest of the networks for all datasets. This occurs for multiple reasons, but the most likely one is related to the results of the second test, which is the detection of different victim body parts.

#### 3.1.3. Class Average Precision for Implemented Neural Network

For this test, the datasets were labeled with five classes: ‘person’, ‘head’, ‘arm’, ‘leg’, and ‘torso’. Due to the similarity of some of these classes to each other, as can be the case for the arms and legs in the thermal images, there is a large increase in classification losses. This same problem is presented to a lesser extent by the Faster R-CNN network but not by the YOLOv3 network.

The inference results are very satisfactory, showing high accuracy and recall and low loss in predictions, making YOLOv3 a viable network for victim detection.

**Figure 12:**
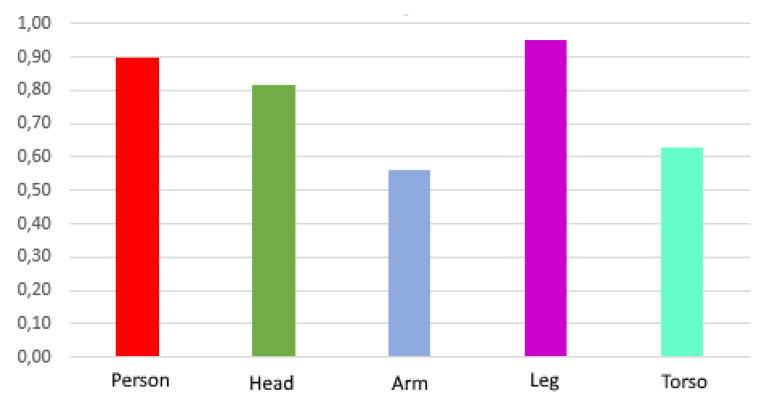
Average class precision for YOLOv3. Source: Authors.

In Figure 12, the average accuracy of each class for the three datasets for YOLOv3 is shown in order to find out which body parts are easier to detect.

As can be seen in Figure 12, all classes have good average precision. The classes ‘person’, ‘head’, and ‘leg’ have a very high accuracy, reaching 0.95 for the case of ‘leg’, while the classes ‘arm’ and ‘torso’ have a somewhat lower accuracy, but within acceptable margins.

The test aimed at evaluating the distinction between normal people and victims was carried out by evaluating different videos featuring both bystanders and alleged victims on the YOLOv3 network. The effectiveness of the method of analyzing the length-to-width ratio of the detected person to distinguish the two cases was evaluated, and the results obtained were more than satisfactory. Each time a possible victim was detected in the image, a message was displayed on the screen alerting of the situation and the coordinates of the image where the victim was located.

### 3.2. Efficiency of Victim Detection Using the Proposed Method in PDE

This test sought to evaluate the performance of the YOLOv3 model trained thermal images captured by the Unitreee Robot. The result was good, although it presented a slightly higher classification error between leg and arm classes than previous tests. Despite this, the model is perfectly valid, detecting everything correctly on most occasions.

**Figure 13:**
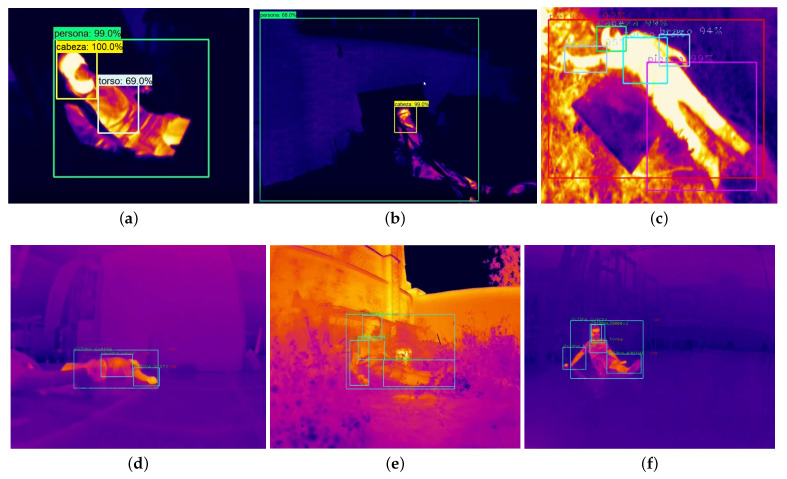
Examples of victim detection with Faster R-CNN, SSD, and YOLOv3. The efficiency in the detection of victims respectively is a = 99%, b = 60%, c = 98%, d = 97%, e = 96%, and f = 99%. (**a**) Faster R-CNN, (**b**) SSD, (**c**) YOLOv3 (Day outdoor), (**d**) YOLOv3 (Day indoor), (**e**) YOLOv3 (Day outdoor), and (**f**) YOLOv3 (Night indoor). Source: Authors.

In this way, the effectiveness of YOLOv3 has also been tested in indoor environments. The main results of the identification of victims are shown in Figure 13, in different environments and lighting conditions, with SSD, R-CNN, and YOLOv3.

Appendix B shows the videos of the real-time execution for detecting victims in the scenarios.

### 3.3. Comparison of Proposed and Traditional Methods for Identifying Victims

**Figure 14:**
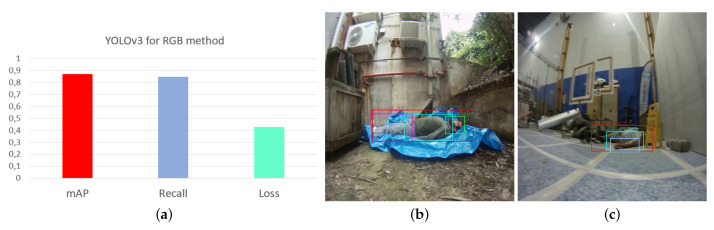
Evaluation of the conventional method that uses RGB images for the detection of victims with good lighting conditions, using CNN-YOLOv3. (**a**) Neural Network Training. (**b**) Outdoor evaluation. (**c**) Indoor evaluation. Source: Authors.

For the development of this comparison, a YOLOv3 neural network has been trained for the detection of victims using RGB images, and the results of the training and the validations are shown in the Figure 14. The same labels used in the thermal method have been used. Results in environments with good light conditions have a high percentage of detection efficiency.

The RGB images used for this method have been captured by the real sense that the robot has in its front part. The procedure for real-time processing is similar to that used in the proposed method.

**Figure 15:**
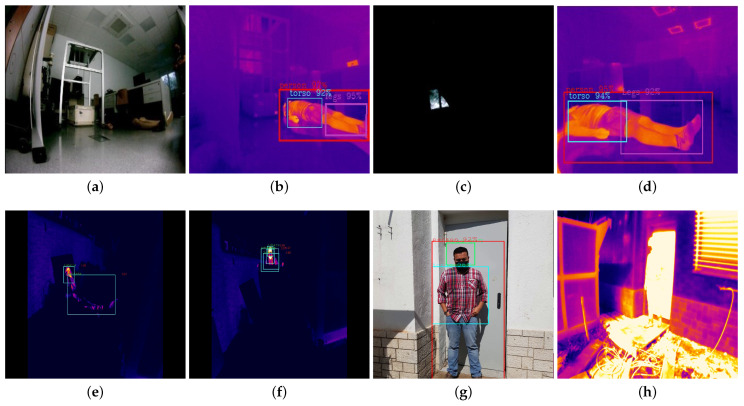
Evaluation of the conventional methods in front of proposed method for victims detection with different lighting conditions, using CNN-YOLOv3. (**a**) Case 1: Bad detection of RGB method in low light. (**b**) Case 1: Good detection of Thermal method in low light. (**c**) Case 2: Bad detection of RGB method in absence of light. (**d**) Case 2: Good detection of Thermal method in absence of light. (**e**) Case 3: Good detection of victims (fully covered) for the thermal method. (**f**) Case 4: Good detection of victims (partially covered) for the thermal method. (**g**) Case 5: Good detection of people in front of heat sources for RGB method. (**h**) Case 5: Bad detection of people in front of heat sources for Thermal method. Source: Authors.

Figure 15 shows different situations analyzed in real environments where both methods have been tested. Figure 15a,b corresponds to the RGB and Thermal methods, respectively, as well as Figure 15c,d. In both cases, the thermal images correspond to the RGB images, however the RGB detection is poor due to the very poor lighting conditions.

Figure 15e,f corresponds to thermal images of totally and partially covered victims, the thermal method for this case due to the emissivity of the covering material (plastic), is the best option.

Finally, Figure 15g,h corresponds to a person in front of a door that has accumulated heat during a summer day (heat source). In this case, the thermal method is more effective.

**Figure 16:**
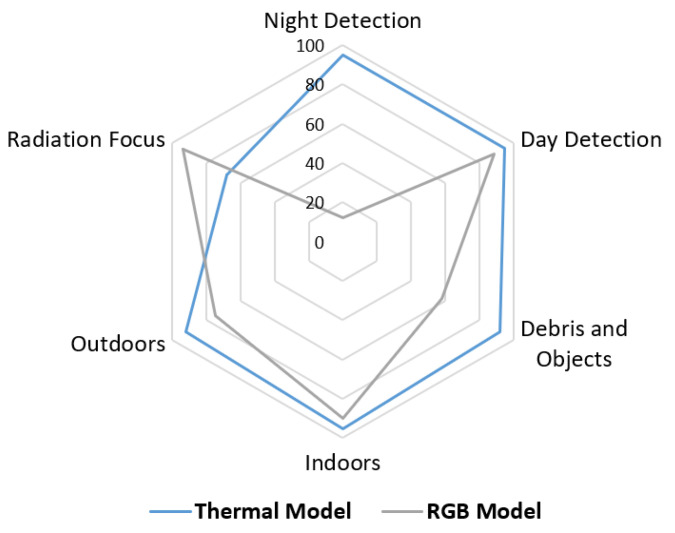
Percentage comparison of efficiency of the analyzed methods (Thermal and RGB). Source: Authors.

Figure 16 shows the radial graph of the percentage parameters obtained from experimental for both methods, where it can be concluded that for most environments the proposed method is more efficient than the conventional ones. On the other hand, one of the shortcomings of this method falls on the presence of large heat sources. Therefore, a solution would be to combine both methods to obtain a more robust system in the face of these disturbances.

For applications such as detecting people in specific places, such as public spaces like streets or PDE. Apart from the disadvantages above, visible spectrum imaging has problems related to the resolution of these cameras required for such a task. Conventional RGB cameras require a much higher image resolution than thermal cameras for the same accuracy and performance.

Using thermal images with a much lower resolution than ordinary cameras, processing and storage in memory require much less computational power. In some situations, for cameras with very low resolution, the computational cost is so low that it can be carried out on embedded systems with very limited resources. In these cases, the energy consumption is also lower.

**Figure 17:**
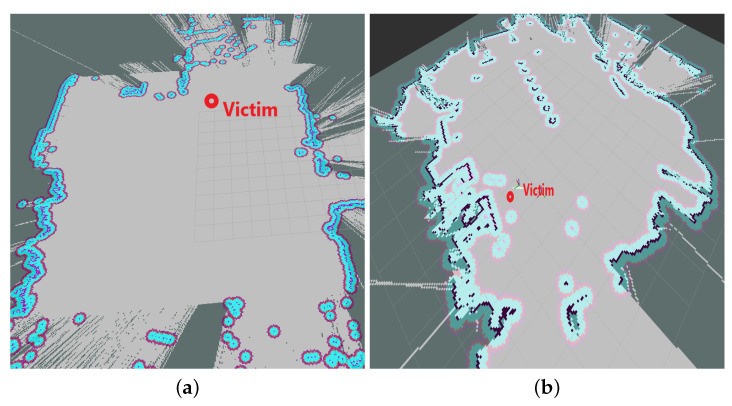
Victims location in the mapped environment. (**a**) Victim detection in reconstructed Scenario 1. (**b**) Victim detection in reconstructed Scenario 2. Source: Authors.

Figure 17 shows the robot exploration result within the explored environment, the victims have been located within the map generated during the exploration of the robot.

## 4. Conclusions

This article shows the proof-of-concept of an integrated Robotic System for victim detection in post-disaster environments using a quadruped robot as a medium to capture and transmit thermal images in real-time. Thermal images were processed through neural networks to analyze the existence of victims in an analyzed environment area. To this end, several subsystems were developed and integrated into ROS. The method was executed and validated on a recreated PDE according to NIST regulation.

The use of the Unitree A1 quadruped robot has allowed the exploration area of the PDE to be overcrowded, thanks to its locomotion system by legs that allow adaptability to uneven floors or with rubble, great agility, and speed for movement. The technology that this robot has, combined with ROS, allowed the development of the application in real-time.

The great advances in computer vision that have taken place in the last few years, due to the incorporation of neural networks into vision models, have meant a great leap forward in the field of computer vision. There are many architectures for neural networks, such as Faster R-CNN, SSD, or YOLO, which implement a series of algorithms capable of performing object detection, each of them having a series of advantages and disadvantages. These three models have been tested in this work to analyze their effectiveness in detecting victims in PDE.

The SSD network has numerous drawbacks for the victim detection task, which cause it to have a high loss value, making its correct application impossible. The Faster R-CNN network greatly improves the results obtained concerning SSD, but its slow inference speed makes it practically impossible to run in real-time.

The YOLOv3 network has a much higher MAP and recall than the other two networks, with values of 85% and 95%, respectively, and a slightly lower loss of approximately 35%. In addition, YOLOv3 has the highest inference speed compared to the other networks. Therefore, it has been concluded that this network is the most suitable one for detecting victims.

The method implemented for the distinction between victim and pedestrians, based on measures the length-to-width ratio of the ‘rescuer’ class to distinguish rescuer from victims, is a viable method that provides satisfactory results.

The effectiveness of the proposed victim detection method has been validated through real-life tests in both indoor and outdoor environments, and for day and night environments it has also shown great efficiency even for materials that cover entirely victims, such as plastics obtaining an efficiency superior to 90% in the detection of victims determined experimentally.

A combination of the RGB method and the proposed method is established to obtain an even more robust system within the lines of future work.

## Figures and Tables

**Table 1:** Evaluation of the neural network parameters.

Component	Amount	Description
Unitree A1	1	Quadruped Robot
Real-Sense	1	RGB-Depth Sensor
Optris Pi640	1	Thermal Camera
Nvidia Jetson Xavier-NX	1	Embedded On-board System
MSI Laptop	1	External Core System

**Table 2:** Elements and components of the mobile platform [35,36].

Material	Temperature (°C)	ϵ
Aluminum, glossy laminated	170	0.04
Asphalt	20	0.93
Concrete	25	0.93
Lead, rusted	20	0.28
Ice	0	0.97
Iron, frosted	20	0.24
Iron, shiny	150	0.13
Iron, rusted	20	0.85
Soil	20	0.66
Glass	90	0.94
Silver	20	0.02
Wood	70	0.94
Plastic (PE, PP, PVC)	20	0.94

## Abbreviations

The following abbreviations are used in this manuscript:

ROS	Robot Operating System
RVIZ	ROS Visualization
CNN	Convolutional Neural Networks
PDE	Post-Disaster Environments
TASAR	Team of Advanced Search And Rescue Robots
RGB	Red Green and Blue

## Appendix A

Datasets for Neural Network Training https://mega.nz/folder/T3BBHSqa#QJkfC2yYp5zPx0OCDP2XqA, accessed on 30 October 2021.

## Appendix B

Videos for Unitree https://mega.nz/folder/GjB3HIKK#qVg7jyOIc55xJU0hqhKXdw, accessed on 30 October 2021.
